# Longitudinal patterns of physical activity, sedentary behavior and sleep in urban South African adolescents, Birth-To-Twenty Plus cohort

**DOI:** 10.1186/s12887-019-1619-z

**Published:** 2019-07-18

**Authors:** Sara K. Hanson, Richard J. Munthali, Lisa K. Micklesfield, Felipe Lobelo, Solveig A. Cunningham, Terryl J. Hartman, Shane A. Norris, Aryeh D. Stein

**Affiliations:** 1 Doctoral Program in Nutrition and Health Sciences, Laney Graduate School, Emory University, Atlanta, GA USA; 2 Medical Research Council of South Africa / University of the Witwatersrand Developmental Pathways for Health Research Unit, Faculty of Health Sciences, University of the Witwatersrand, Johannesburg, South Africa; 3 Hubert Department of Global Health, Rollins School of Public Health, Emory University, Atlanta, GA USA; 4 Department of Epidemiology, Rollins School of Public Health, Emory University, Atlanta, GA USA

**Keywords:** Adolescence, Low or middle income country, Physical activity, Sedentary behavior, Sleep

## Abstract

**Background:**

Adolescence is a critical phase of human development that lays the foundation for health in later life. Of the 1.8 billion adolescents in the world, roughly 90% live in low and middle-income countries. Yet most longitudinal studies of adolescent physical activity, sedentary behavior, and sleep come from high-income countries. There is a need for a better understanding of these behaviors to inform obesity and chronic disease prevention strategies.

**Aims:**

The aim of this study is to identify longitudinal patterns and associations between physical activity, sedentary behavior and sleep in urban South African adolescents.

**Methods:**

We analyzed data from the Birth-to-Twenty Plus Cohort (Bt20+), a longitudinal study of children in Soweto, Johannesburg, South Africa. Behaviors were self-reported annually between ages 12 and 17 y. We used Latent Class Growth Analysis to group participants into classes based on common longitudinal trajectories of time spent in informal physical activity, organized sports, walking to and from school, sedentary behavior, and school-night and weekend sleep, respectively. We performed group-based multi-trajectory modeling to identify latent clusters of individuals who followed similar trajectories of informal physical activity, organized sports and walking to and from school, and who followed similar trajectories of these three domains together with sedentary behavior and sleep.

**Results:**

The large majority of males (82%) and all females failed to meet the World Health Organization (WHO) physical activity recommendation for adolescents of 60 min of moderate-vigorous intensity physical activity per day. The physical activity domains clustered together in three multi-trajectory groups that define individuals’ overall physical activity pattern. While two patterns indicated decreases in physical activity throughout adolescence, one pattern, including 29% of the sample in males and 17% of the sample in females, indicated higher levels of activity throughout adolescence. Sedentary behavior and sleep trajectories did not cluster together with the physical activity domains.

**Conclusion:**

Most adolescents in this South African population did not meet WHO recommendations for physical activity. In this population, trajectories of sedentary behavior and sleep were independent of physical activity.

**Electronic supplementary material:**

The online version of this article (10.1186/s12887-019-1619-z) contains supplementary material, which is available to authorized users.

## Background

Adolescence is a critical phase of human development between childhood and adulthood that lays the foundation for later life health [1, 2]. The current generation of people aged 10–24 years is the largest in human history and makes up nearly a quarter of the world’s population. [3] However, because adolescents typically have low mortality and chronic disease rates, this population has until recently been relatively neglected in global health research and policy [4, 5]. The recent releases of the World Health Organization (WHO) Recommendations on Adolescent Health and the Lancet Commission on Adolescent Health and Wellbeing have focused interest on adolescent health [6, 7].

Health-related risk behaviors (e.g., physical activity, dietary habits, initiation of smoking) adopted during adolescence can have substantial effects on later health, including increased risk of obesity, coronary heart disease, diabetes, some cancers and premature death [8–10]. Globally, an estimated 80% of 13–15 year olds do not meet the current WHO physical activity recommendation of 60 min of moderate to vigorous intensity physical activity per day [6, 9, 11]. In the last decade, sedentary behavior has been identified as its own health risk, independent of physical activity [12, 13], and the majority of children and adolescents exceed the recommendations of 2 h or less of screen time daily [14–16]. Insufficient sleep in adolescence is recognized as a serious health risk, yet data from multiple countries suggest that most adolescents are sleeping fewer hours than the 8–10 h that they need [17–20]. There is increasing interest in considering the set of movement behaviors as whole rather than considering them separately [21, 22].

Of the 1.8 billion adolescents in the world, roughly 90% live in low- or middle-income countries (LMICs) [3, 4]. Many LMICs are experiencing an epidemiological transition characterized by a shifting burden from infectious to non-communicable diseases [23]. As the burden of non-communicable diseases in LMICs continues to rise, there is a need for research on modifiable risk factors that could better inform prevention strategies [24]. However, longitudinal data on modifiable risk factors spanning adolescence are rare. Specifically, the Lancet Physical Activity Series Working Group highlights a severe lack of data from Africa [11, 20].

Since South Africa’s political transformation in 1994, the country has undergone rapid urbanization, globalization and socioeconomic change [25]. An increasing prevalence of overweight and obesity and shifts in dietary patterns in urban South African youth have been observed [26, 27]. Patterns of physical activity, sedentary behavior and sleep may be contributing to the high prevalence of overweight and obesity, but these behaviors in urban South African adolescents remain unexplored. Therefore, the aims of this study are to describe longitudinal patterns of physical activity, sedentary behavior and sleep in urban South African adolescents and identify factors that predict these patterns.

## Methods

### Study sample

We analyzed data from participants in the Birth-to-Twenty Plus Cohort (Bt20+), a longitudinal study of singleton children born between April and June 1990 in Soweto and in Johannesburg, South Africa. Detailed information on the cohort is provided elsewhere [28, 29]. Singleton children born in public hospitals to Soweto or Johannesburg residents and residing in the area for at least 6 months after the birth were eligible for inclusion. Of all 5449 births notified in the study area, 3,273 met the residency eligibility criterion and were recruited. Most attrition occurred in the first few years of the study, primarily due to migration out of the study area [30]. At inception the cohort was 78% Black African, with approximately equal numbers of males (49%) and females (51%); attrition was higher for White cohort members [31]. Ethical approval was obtained from the University of the Witwatersrand Committee for Research on Human Subjects (approval ID #M010556). Written participant assent and caregiver permission to participate in the study were obtained at each wave.

### Assessment of movement behaviors

Physical activity and sedentary behavior were self-reported at ages 12, 13, 14, 15, 16 and 17/18 years using a questionnaire developed for South African children and validated in a South African population (Additional file 1) [32]. Respondents were asked to report about frequency and duration of informal physical activity, physical education at school, school sports, club sports and active transportation on a typical week over the past 12 months. Informal physical activity includes physical activity during school breaks or outside of school and not part of a sports team or club, such as skipping, traditional games and playing with a ball; respondents were asked to report on the duration of the three most frequent activities. Due to changes in the wording of the questionnaire between age 12 and age 13, we did not include informal physical activity at age 12 in our analyses. Physical education was defined as any exercise class supervised by a teacher during school time. School sport was defined as any extramural sport organized by the school; club sport was defined as any private extramural sport. Active transportation was defined as walking or biking to and from school; as few participants reported using bicycles, we included only time spent walking in our analyses. Questions regarding sedentary behavior included time spent watching television and videos; reading, drawing and homework; playing a musical instrument; playing video/computer games, and internet surfing, before and after school. At ages 13, 14, 15, 16 and 17/18 years, respondents reported the times they went to bed and woke up on school nights and weekends/holidays, from which we calculated hours of sleep. No information was solicited on quality of sleep.

We calculated informal physical activity minutes by summing the minutes spent per week in each of the three most frequent activities. We calculated physical education minutes by multiplying the reported number of classes per week by the reported number of minutes per class. We created a composite measure of organized sport by combining reported minutes per week of school sports and club sports. We inferred duration of competition for each sport based on sport-specific rules (e.g., rugby has two 40-min halves). If a sport had no rules governing the duration of competition (e.g., gymnastics or tennis), we inferred a duration of 60 min. We then summed the reported number of minutes per week across all reported sports. To better represent moderate-vigorous intensity physical activity (MVPA) participation, only sports with a metabolic equivalent (MET) value ≥3 based on the Compendium of Physical Activity for Youth were included [33]. Walking minutes per week was calculated as the sum of minutes per day spent walking to and from school, multiplied by 5 days per week. Intensity of walking was not reported, and we did not include this walking in our estimate of MVPA. We calculated total sedentary behavior minutes per week by summing the reported time spent per day in each of the activities.

### Predictors of movement behaviors

We selected potential predictors a priori, including factors at the individual, maternal and household level [34–37]. All these variables were collected by questionnaire administered to the mother at recruitment into the cohort or at child age 7/8 y. Covariates include socio-economic status (SES) at child age 7/8 years, mother’s schooling at child age 7/8 y (matriculated from secondary school, did not matriculate), mother’s marital/union status at child age 7/8 y (in union, not in union) and whether the child was first-born or not. These variables have been identified as correlates of physical activity and sedentary behavior in childhood in a subset of Bt20+ participants and in adolescence in rural South Africa [38, 39]. Household physical assets at age 7/8 were used as a proxy for socio-economic status (SES). A validated standardized questionnaire based on the Demographic and Health survey for developing countries was used to calculate an asset score [40]. Mothers were asked about home type, home ownership, electricity in the home, and ownership of a television, car, refrigerator, washing machine, and phone. The resulting score was categorized into quintiles.

### Inclusion and exclusion

We restricted analyses to participants who were Black African due to low numbers of the other ethnic groups. Analyses were further restricted to participants who had physical activity, sedentary behavior and sleep data for at least two data points between the ages of 12 and 17/18 years to ensure model stability.

### Statistical analyses

We stratified all analyses by sex due to well-established sex differences in adolescent physical activity levels [11, 39, 41, 42]. We conducted a sensitivity analysis by comparing SES asset quintile, mother’s education, mother’s marital /union status, mother’s age at delivery, and parity, between participants who met inclusion criteria (Black African ethnicity and ≥ 2 data points) and participants who did not meet inclusion criteria, using chi-square tests. Descriptive summary data are presented as 25th percentile (Q1), median (Q2) and 75th percentile (Q3) for time spent in physical activity, sedentary behavior and sleep (Table 1).

**Table 1:** Characteristics of the study sample by sex, Birth-to-Twenty Plus cohort, Johannesburg, South Africa

		Males			Females		
		Included^a^	Excluded	*P*-value	Included	Excluded	*P*-value
N		669	577		745	577	
SES-asset quintile, %^b^	1 (lowest)	17	22	0.19	22	28	0.19
	2	20	19		18	22	
	3	30	31		29	25	
	4	22	16		19	15	
	5 (highest)	11	12		12	10	
Mother had matriculated from secondary school, %^b^		29	25	0.24	31	30	0.74
Mother married or in union, %^b^		29	36	0.32	36	44	0.04
Mother’s age at delivery, %	< 25 y	48	47	0.24	47	43	0.28
	25–29 y	23	27		24	28	
	≥30 y	29	25		29	28	
Child was first-born, %		38	33	0.05	41	32	0.001

^a^Among participants who are of Black African ethnicity, provided activity data at ≥2 time points

^b^Ascertained by interview of the mother at child age 7/8 y

We used Latent Class Growth Analysis (LCGA) to group participants into distinct classes based on common longitudinal trajectories of informal physical activity, physical education, organized sports, walking to and from school, sedentary behavior, school-night sleep or weekend sleep. LCGA is a type of Growth Mixture Modeling where it is assumed that the estimated variance and covariance of the growth factors within each class are fixed to zero [43]. We used full information maximum likelihood estimation to integrate all available information based on missing at random assumptions [44].

We determined the optimal number of latent classes using Bayesian Information Criterion (BIC), entropy, the Lo-Medell-Rubin-Likelihood Ratio Test (LMR-LRT) and the Bootstrap Likelihood Ratio Test (BLRT) [43]. The best model was considered the one with the lowest BIC value, highest entropy significant LMR-LRT, and significant BLRT. In addition to model fit indices, we also considered the percentage of participants per class, parsimony, theoretical justification and interpretability [43]. We implemented a restriction requiring at least 5% per class to avoid small group sizes. After establishing the number of trajectory classes, we assigned participants to a class based on the individual’s maximum posterior probability. Because we determined there to be only one physical education trajectory class in both males and females, we did not include this domain in subsequent analyses. We explored the relationships between individual trajectory classes across domains using chi-square tests. We identified predictors of trajectory class membership for each movement behavior using multinomial logistic regression. We conducted the LCGA in MPlus 7.3 [44] and the logistic regression in Stata version 14. We set significance at *p* < 0.05.

To describe overall movement behavior patterns, we performed multi-trajectory modeling. This modeling technique is designed to identify latent clusters, or multi-trajectory “groups,” of individuals following similar trajectories over multiple indicators of an outcome [45]. We examined two sets of multi-trajectory models: (1) overall physical activity - informal physical activity, organized sports and walking to and from school; and (2) overall movement behavior defined by the 3 physical activity domains, sedentary behavior and school-night sleep. For both overall physical activity and overall movement behavior, we chose the preferred multi-trajectory model using a two-stage model selection process. In the first stage, we determined the number of multi-trajectory groups based on BIC model fit statistics, practical interest and ability to distinguish between groups. In the second stage, we determined the preferred order of the polynomial defining the shape of each trajectory, given the number of groups selected in the first stage [46]. We compared characteristics of the multi-trajectory groups using chi-square tests, and identified predictors of multi-trajectory group membership using multinomial logistic regression. We conducted the multi-trajectory analysis using a plug-in for Stata version 14 [45].

## Results

These analyses included 1,414 participants (of whom 745 were female), representing 43% of the source population. A flow chart depicting the final sample of eligible participants included in the analyses is provided in Additional file 2: Figure S1. Based on information collected at age 7/8 years, 29% of males and 31% of females had mothers who matriculated from secondary school, 29% of males and 36% of females had mothers who were married/in a union and 38% of males and 41% of females were firstborn (Table 1). There were no significant differences in study characteristics between males included in the analytic sample and males excluded for lack of movement behavior data. Among females, there was a significantly higher percentage of mothers in a union at child age 7/8 years, and a significantly lower percentage of first-born children in those who were excluded compared to those who were included.

### Identification of trajectories

Duration of all forms of physical activity decreased over adolescence, while sedentary behavior increased and duration of weekend sleep remained stable, in both males and females (Table 2). School night sleep decreased across adolescence in males but remained stable in females.

**Table 2:** Duration of physical activity, sedentary behavior and sleep behaviors through adolescence, by sex, Birth-to-Twenty Plus cohort, Johannesburg, South Africa

	12 y				13 y				14 y				15 y				16 y				17 y			
	Any	Q1^a^	Q2	Q3	Any	Q1	Q2	Q3	Any	Q1	Q2	Q3	Any	Q1	Q2	Q3	Any	Q1	Q2	Q3	Any	Q1	Q2	Q3
	%	Min/ wk	Min/ wk	Min/ wk	%	Min/ wk	Min/ wk	Min/ wk	%	Min/ wk	Min/ wk	Min/ wk	%	Min/ wk	Min/ wk	Min/ wk	%	Min/ wk	Min/ wk	Min/ wk	%	Min/ wk	Min/ wk	Min/ wk
Males	*N* = 502				*N* = 669				*N* = 634				*N* = 640				*N* = 660				N = 640			
Informal physical activity^b^	---^3^	–	–	–	94	210	360	638	92	180	360	615	82	60	210	480	51	0	30	240	59	0	120	360
Physical education^b^	49	0	0	150	27	0	0	30	22	0	0	0	15	0	0	0	35	0	0	60	38	45	90	120
Organized sports^b^	84	46	186	383	81	37	138	355	66	0	92	300	46	0	0	245	41	0	0	138	43	0	0	174
Walking to/from school^b^	76	10	150	250	75	0	150	250	81	50	150	250	87	50	150	200	77	20	100	200	70	0	100	200
Sedentary behavior ^b^	100	540	750	750	100	660	990	1500	100	660	990	1680	100	990	1470	1950	100	840	1335	2040	100	1050	1633	2310
School-night sleep^c^	–	–	–	–	–	–	–	–	100	480	540	570	100	480	510	570	100	480	510	540	100	450	480	540
Weekend sleep^c^	–	–	–	–	–	–	–	–	100	540	600	630	100	540	600	660	100	540	600	660	100	540	600	660
Females	*N* = 578				*N* = 745				*N* = 705				N = 705				*N* = 735				*N* = 702			
Informal physical activity^b^	–	–	–	–	90	150	275	510	82	60	210	300	62	0	60	240	30	0	0	45	21	0	0	0
Physical education^b^	47	0	0	120	35	0	0	60	25	0	0	60	20	0	0	0	36	0	0	60	31	60	90	135
Organized sports^b^	73	0	55	149	69	0	55	148	49	0	0	94	15	0	0	0	26	0	0	9	22	0	0	0
Walking to/from school^b^	72	0	150	263	72	0	150	300	75	10	150	300	78	30	150	300	67	0	100	200	57	0	70	225
Sedentary behavior^c^	100	540	780	1080	100%	750	1080	1680	100	660	1020	1680	100	1110	1740	2370	100	1020	1655	2340	100	1260	1950	2790
School-night sleep^c^	–	–	–	–	–	–	–	–	100	480	510	540	100	450	510	540	100	459	510	520	100	420	480	540
Weekend sleep4^c^	–	–	–	–	–	–	–	–	100	540	600	630	100	540	600	660	100	540	600	660	100	540	600	660

^1^Respondents reporting at least 1 min per week of specific behavior

^a^Q1: 25th percentile; Q2: 50th percentile; Q3: 75th percentile

^b^Minutes per week

^c^Minutes per night

-- Module not administered

**Fig. 1:**
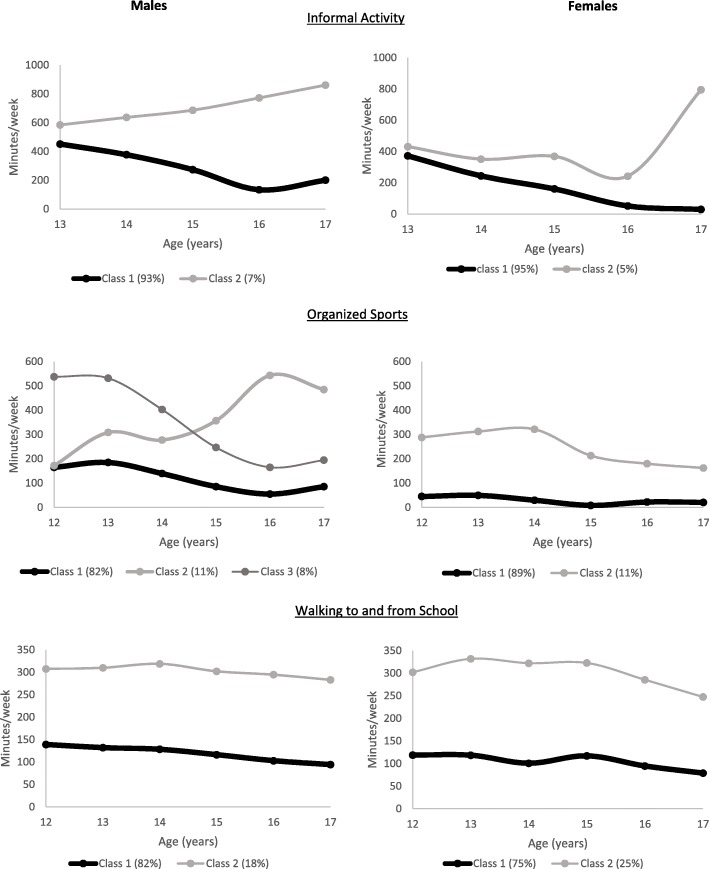
Physical activity trajectories through adolescence, by domain and sex, Birth to Twenty Plus Cohort, Johannesburg, South Africa

We identified 2 trajectories for informal physical activity for males, with one (93% of the sample) showing a steady decrease in physical activity from age 13–18 years and the other showing a steady increase (Fig. 1). In females, we also identified two informal physical activity trajectories with one (95% of the sample) showing a steady decrease and the other showing a decrease followed by a sharp increase at age 16 years. For organized sports, we identified 3 trajectories for males: consistently low (82%), increasing (11%) and decreasing (8%) and two for females, representing no participation (89%) or some participation in organized sports from age 12. We identified 2 trajectories of walking to and from school, lower (representing 82 and 75% of males and females, respectively) and higher.

**Fig. 2:**
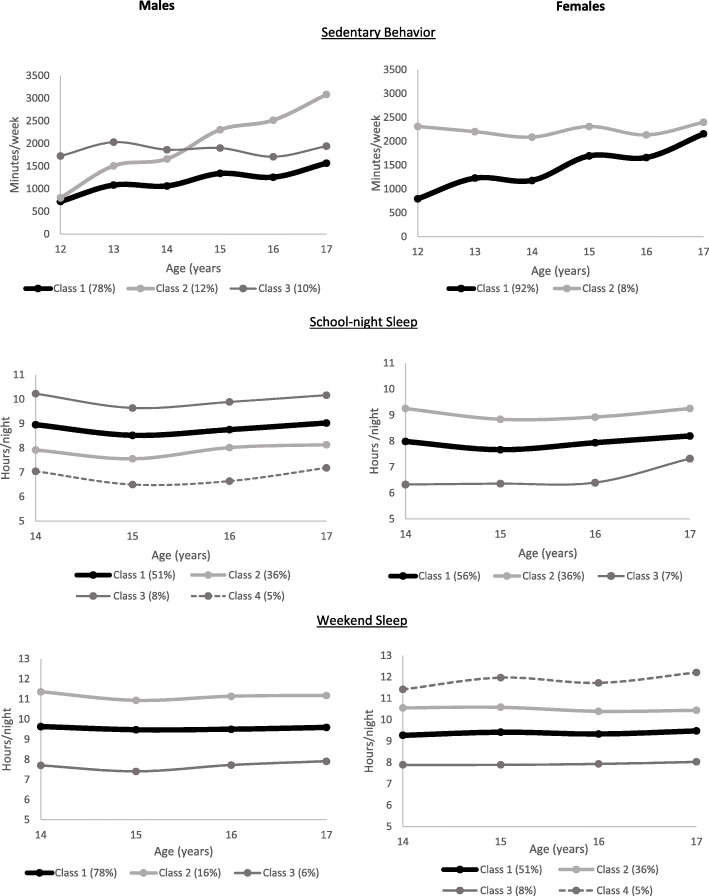
Sedentary behavior and sleep trajectories through adolescence, by sex, Birth to Twenty Plus Cohort, Johannesburg, South Africa

For sedentary behavior, we identified 3 trajectories in males, showing steady lower (78%), steady higher (10%) and increasing (12%) and 2 trajectories in females, increasing (92%) and steady higher (Fig. 2). For school-night sleep, we identified 4 trajectories in males and 3 trajectories in females. For weekend sleep, we identified 3 trajectories for males and 4 trajectories for females. Model fit statistics of individual trajectories are provided in Additional file 2: Tables S1 and S2.

### Associations between trajectories

In males, the distribution of the sedentary behavior trajectories was independent of the distributions of all the physical activity domain trajectories (Additional file 2: Table S3). In females, the distribution of the sedentary behavior trajectories differed across the distribution of the walking to and from school trajectories (*p* = 0.04) but was independent of the distributions of the informal physical activity and organized sport trajectories (Additional file 2: Table S4). In both males and females, the distributions of school-night sleep trajectories and weekend sleep trajectories differed across the distribution of sedentary behavior trajectories (*p* < 0.001 and *p* = 0.002). The distribution of the school-night sleep trajectories differed by the distribution of the walking to and from school trajectory in females but not in males (*p* = 0.001) (Additional file 2: Tables S5 and S6).

### Predictors of trajectory class membership

Informal physical activity: Being in a higher SES quintile was associated with higher likelihood of being in the steady increasing trajectory compared to the steady decreasing trajectory among males (Relative Risk Ratio (RRR) =1.48 95% CI: 1.11–1.98) (Table 3).

**Table 3:** Relative risk ratios and 95% confidence intervals for predictors of trajectory class membership, Birth-to-Twenty+

Males		Females	
	RRR (95% CI)		RRR (95% CI)
Informal Physical Activity			
Class 1--- decreasing (ref)		Class 1--- decreasing (ref)	
Class 2--- increasing		Class 2--- decreasing then increase at 16 y	
SES (asset quintile)	1.48 (1.11–1.98)	SES (asset quintile)	0.95 (0.71–1.29)
mother’s schooling	0.59 (0.26–1.34)	mother’s schooling	1.04 (0.45–2.43)
mother’s marriage/union status	0.48 (0.23–1.01)	mother’s marriage/union status	0.71 (0.32–1.58)
parity	0.59 (0.28–1.24)	parity	0.70 (0.32–1.52)
Pseudo R^2^	0.04	Pseudo R^2^	0.006
Organized Sports			
Class 1--- consistently low (ref)		Class 1--- no participation (ref)	
Class 2--- increasing		Class 2--- some participation	
SES (asset quintile)	0.86 (0.68–1.10)	SES (asset quintile)	1.04 (0.84–1.30)
mother’s schooling	1.53 (0.81–2.87)	mother’s schooling	0.93 (0.51–1.71)
mother’s marriage/union status	1.15 (0.63–2.10)	mother’s marriage/union status	0.76 (0.42–1.36)
parity	1.25 (0.69–2.29)	parity	1.07 (0.62–1.86)
Class 3--- decreasing			
SES (asset quintile)	1.00 (0.75–1.33)		
mother’s schooling	1.01 (0.48–2.14)		
mother’s marriage/union status	0.87 (0.43–1.75)		
parity	1.31 (0.66–2.61)		
Pseudo R^2^	0.007	Pseudo R^2^	0.003
Walking to and from school			
Class 1--- lower (ref)		Class 1--- lower (ref)	
Class 2--- higher		Class 2--- higher	
SES (asset quintile)	0.81 (0.66–0.98)	SES (asset quintile)	0.68 (0.58–0.81)
mother’s schooling	0.55 (0.31–0.99)	mother’s schooling	0.47 (0.28–0.80)
mother’s marriage/union status	0.70 (0.43–1.14)	mother’s marriage/union status	1.12 (0.73–1.72)
parity	0.69 (0.42–1.14)	parity	0.84 (0.55–1.29)
Pseudo R^2^	0.04	Pseudo R^2^	0.07
Sedentary Behavior			
Class 1--- stable lower (ref)		Class 1--- increasing (ref)	
Class 2--- increasing’		Class 2--- stable higher	
SES (asset quintile)	1.08 (0.86–1.36)	SES (asset quintile)	1.29 (0.99–1.67)
mother’s schooling	1.64 (0.91–2.96)	mother’s schooling	1.50 (0.76–2.94)
mother’s marriage/union status	1.46 (0.83–2.56)	mother’s marriage/union status	1.18 (0.62–2.25)
parity	1.11 (0.63–1.98)	parity	0.78 (0.40–1.51)
Class 3--- stable higher			
SES (asset quintile)	1.06 (0.85–1.34)		
mother’s schooling	1.82 (1.02–3.25)		
mother’s marriage/union status	1.52 (0.87–2.65)		
parity	1.14 (0.76–2.35)		
Pseudo R^2^	0.02	Pseudo R^2^	0.03
School-night Sleep			
Class 1--- 9 h/night (ref)		Class 1---8 h/night (ref)	
Class 2--- 8 h/night		Class 2---9 h/night	
SES (asset quintile)	1.10 (0.93–1.29)	SES (asset quintile)	0.82 (0.70–0.95)
mother’s schooling	2.43 (1.58–3.74)	mother’s schooling	0.46 (0.30–0.72)
mother’s marriage/union status	1.35 (0.90–2.02)	mother’s marriage/union status	0.80 (0.54–1.18)
parity	0.70 (0.46–1.07)	parity	0.90 (0.62–1.32)
Class 3--- 10 h/night		Class 3--- 6.5 h/night	
SES (asset quintile)	0.79 (0.59–1.04)	SES (asset quintile)	1.11 (0.84–1.45)
mother’s schooling	0.55 (0.20–1.50)	mother’s schooling	1.18 (0.59–2.32)
mother’s marriage/union status	0.76 (0.38–1.53)	mother’s marriage/union status	1.10 (0.54–2.13)
parity	0.49 (0.23–1.02)	parity	1.81 (0.93–3.52)
Class 4--- 7 h/night			
SES (asset quintile)	1.40 (0.95–2.06)		
mother’s schooling	3.63 (1.47–8.98)		
mother’s marriage/union status	1.32 (0.55–3.17)		
parity	1.19 (0.49–2.90)		
Pseudo R^2^	0.05	Pseudo R^2^	0.04
Weekend Sleep			
Class 1--- 9.5 h/night (ref)		Class 1--- 9 h/ night (ref)	
Class 2--- 11 h/night		Class 2---10 h/night	
SES (asset quintile)	1.02 (0.83–1.24)	SES (asset quintile)	1.01 (0.87–1.17)
mother’s schooling	0.56 (0.32–1.05)	mother’s schooling	1.07 (0.71–1.62)
mother’s marriage/union status	0.82 (0.50–1.25)	mother’s marriage/union status	1.14 (0.78–1.68)
parity	0.65 (0.39–1.09)	parity	1.06 (0.73–1.54)
Class 3--- 7.5 h/night		Class 3--- 8 h/night	
SES (asset quintile)	1.30 (0.95–1.79)	SES (asset quintile)	1.10 (0.86–1.43)
mother’s schooling	0.97 (0.43–2.16)	mother’s schooling	1.38 (0.67–2.78)
mother’s marriage/union status	1.00 (0.47–2.13)	mother’s marriage/union status	1.16 (0.60–2.26)
parity	0.92 (0.43–1.97)	parity	0.90 (0.46–1.74)
		Class 4--- 11 h/night	
		SES (asset quintile)	1.04 (0.76–1.43)
		mother’s schooling	1.53 (0.64–3.65)
		mother’s marriage/union status	0.87 (0.38–1.97)
		parity	0.42 (0.17–1.02)
Pseudo R^2^	0.02	Pseudo R^2^	0.006

Walking to and from school: Being in a higher SES quintile and having a mother who matriculated from secondary school were each associated with lower likelihood of being in the higher trajectory compared to the lower trajectory in males (RRR = 0.81 95% CI: 0.66–0.98 and RRR = 0.55 95% CI: 0.33–0.99) and females (RRR = 0.68 95% CI 0.58–0.81 and RRR = 0.47 95% CI: 0.28–0.80).

Sedentary behaviors: Among males, having a mother who matriculated from secondary school was associated with higher likelihood of being in the increasing trajectory compared to the steady lower trajectory (RRR = 1.82 95% CI: 1.02–3.25).

Sleep: In adjusted models, having a mother who matriculated from secondary school was associated with over two times the likelihood of being in the 8 h per night trajectory and over three times the likelihood of being in the 7 h per night trajectory compared to the 9 h per night trajectory in males and lower likelihood of being in the 9 h per night trajectory compared to the 8 h per night trajectory (RRR = 0.82 95% CI: 0.70–0.95 and RRR = 0.46 95% CI: 0.30–0.72) in females.

None of these variables were associated with informal physical activity or sedentary behavior trajectories in females or with organized sport and weekend sleep trajectories in either males or females.

### Multi-trajectory modeling

Overall physical activity: We identified 3 multi-trajectory groups for males and for females (Additional file 2: Figure S2). In both male and female multi-trajectory groups 1 and 3, informal physical activity and organized sport trajectories declined throughout adolescence. The main distinguishing characteristic between groups 1 and 3 was the level of walking to and from school. Group 2 maintained higher levels of both informal physical activity and organized sports than groups 1 and 3 and had a walking to and from school trajectory similar to group 3. Group 1, representing 23% of the sample in males and 32% of the sample in females, respectively, can be described as “Decreasing Activity, non-walkers”, group 2 (29 and 17%, respectively) as “Active, walkers” and group 3 (48 and 51%, respectively) as “Decreasing activity, walkers.” Model fit statistics of the physical activity multi-trajectory groups are shown in Additional file 2: Table S7.

Higher SES quintile and having a mother who matriculated from secondary school were each associated with increased likelihood of being in group 1 (“Decreasing Activity, non-walkers”) compared to group 3 (“Active, walkers”) but were not associated with risk of being in group 3 (“Decreasing activity, walkers” compared to group 2 in either males or females (Table 4).

**Table 4:** Relative risk ratios and 95% confidence intervals for predictors of physical activity^a^ multi-trajectory groups, Birth-to-Twenty+

	Males			Females		
	Group 1 “Decreasing activity, non-walkers”	Group 2 “Active, walkers”	Group 3 “Decreasing activity, walkers”	Group 1 “Decreasing activity, non-walkers”	Group 2 “Active, walkers”	Group 3 “Decreasing activity, walkers”
	RRR (95% CI)	RRR (95% CI)	(ref)	RRR (95% CI)	RRR (95% CI)	(ref)
SES (asset quintile)	1.48 (1.22–1.81)	1.04(0.87–1.23)	(ref)	1.80 (1.51–2.14)	1.16 (0.95–1.42)	(ref)
Mother’s schooling	2.39 (1.47–3.88)	1.21 (0.75–1.96)	(ref)	3.43 (2.21–5.32)	1.91 (1.08–3.37)	(ref)
Mother’s marital status	1.69 (1.06–2.70)	1.21 (0.79–1.87)	(ref)	0.89 (0.57–1.38)	0.57 (0.33–0.97)	(ref)
Mother’s parity	1.49 (0.93–2.39)	1.24 (0.81–1.92)	(ref)	0.94 (0.61–1.45)	0.58 (0.34–0.97)	(ref)
Pseudo R^2^			0.05			0.11

^a^Overall physical activity includes informal physical activity, organized sports and walking to and from school

### Multi-trajectory modeling of all movement behaviors

Overall movement behaviors: We identified 4 multi-trajectory groups for males and 2 for females (Additional file 2: Figure S3). Model fit statistics are shown in Additional file 2: Table S8. In males, the most notable distinctions between the multi-trajectory groups were seen among the 3 physical activity trajectories, with the sedentary behavior and sleep trajectories in each group being nearly identical. In females, the primary distinction between multi-trajectory groups related to walking to and from school, with the other trajectories showing minimal differences between the two groups.

## Discussion

Given the increasing prevalence of overweight and obesity throughout childhood and adolescence in South Africa [26], there has been interest in exploring modifiable lifestyle factors. This study identified distinct sex-specific trajectories of movement behaviors in a cohort of urban South African adolescents.

The patterns of overall physical activity trends, as described by the physical activity multi-trajectory groups, were similar between males and females in this study population, though the amounts of both informal physical activity and organized sports were higher in males. Overall physical activity decreased from age 12 to 17 years. This is consistent with findings from high-income countries [42]. However, we identified groups of both males and females (29 and 17%, respectively) who showed a pattern of maintaining physical activity across adolescence. Understanding what sets these individuals apart may provide valuable information for intervention research and forms the basis for future research.

The WHO recommends that adolescents get 60 min of moderate to vigorous intensity physical activity per day [6]. In our study, organized sports can be considered as a proxy for MVPA, and 82% of males and 100% of females failed to meet these recommendations throughout adolescence. An additional 8% of males met the recommendation in early adolescence but then declined below the recommendation by age 14 years. In contrast, 11% of males did not meet the recommendation in early adolescence but increased their activity to meet the recommendation after age 15 years. Among females, 89% did not participate in any organized sports throughout adolescence. Even in the remaining 11% who did participate in organized sports, none met the recommended duration.

Physical activity behavior is multi-dimensional and influenced by a diverse set of factors [47]. Among the males in our study, participants in a higher asset quintile (a proxy for SES) were more likely to be in the increasing informal physical activity trajectory than the decreasing trajectory. This association was not seen in females. Male adolescents from higher SES families may have fewer household responsibilities than males from lower SES families and females, giving them more free time for play and leisure-time activities. Both males and females from a higher asset quintile were more likely to be in the trajectory that represented lower walking to and from school, which may be indicative of family car ownership or greater access to transportation options. Asset quintile was not related to categorization into organized sports trajectory in either males or females. Micklesfield et al. in rural South African adolescents and McVeigh et al. in a younger subset of this cohort, reported that lower SES was associated with less time spent in school and club sports [38, 39]. Older youth living in an urban setting may have more access to school and club sports than the rural and younger participants in the previously described studies.

Research and public health interventions among adolescents have typically focused on increasing physical activity [48]. However, higher sedentary behavior (i.e. too much sitting), independent of physical activity, is associated with adverse health outcomes [49]. In our study, every sedentary behavior trajectory showed sedentary behavior durations over 2 h per day.

Adolescent sedentary behaviors have been shown to track over time, but most previous studies have used methods that did not assume population heterogeneity [50, 51]. Two studies from high –income country settings that followed participants from ages 5 to 19/20 years demonstrated that sedentary behavior was stable in some participants during the adolescent years of the studies but increased or decreased over time in others [52, 53]. We found a similar pattern in this South African birth cohort, with trajectories of both stable and increasing sedentary time in males and females. We however did not observe a trajectory that decreased over time.

Adolescent sleep has been recognized as a public health concern, with many countries reporting high prevalence of sleep disturbance among youth [20], but little is known about the sleep patterns of adolescents in Africa. In our study, all but the smallest trajectories of males (5%) and females (7%) met the National Sleep Foundation’s guideline for adolescents of 8–10 h of sleep per day [54], but we lack information about the quality of this sleep. In contrast, a 2002 cross-sectional study by Reid et al. in urban South African adolescents reported a total sleep time of just over 7 h, high percentages of daytime napping and sleepiness during school, suggesting insufficient sleep [55]. Unlike most studies of adolescent sleep from around the world, we did not find a decline in sleep time through adolescence [20, 56].

Prior research has found a positive association between SES and sedentary behavior in LMICs [38, 39, 57]. We found no association between asset quintile and sedentary behavior trajectories, but that males whose mothers matriculated from secondary school had higher likelihood of being in the steeper increasing trajectory for sedentary time compared to the consistently lower trajectory. Additionally, we found that higher maternal schooling was associated with being in the lower school-night sleep trajectories in both males and females, consistent with other findings in a low-income setting [58]. Adolescents with more educated parents may be more likely to have smartphones and computers with access to Wi-Fi. They may also receive more academic pressure at home, and therefore may spend more time doing homework, a component of sedentary behavior. Further, these adolescents may delay bedtime trying to complete homework and therefore sleep for a shorter duration.

To our knowledge, this is the first study to use multi-trajectory modeling to represent the associations between multiple physical activity domains in adolescents. This approach allowed us to identify domain-specific patterns while still assessing overall physical activity. In both males and females, those who decreased time spent in organized sports throughout adolescence also decreased informal physical activity, and those who maintained higher levels of organized sports maintained higher levels of informal physical activity. This finding adds to the evidence that organized sport participants are more physically active overall than non-participants and supports the need for strategies to increase and maintain organized sport participation rates [59, 60].

Consistent with our single trajectory analysis, adolescents with higher SES were more likely to be in the “decreasing activity, non-walkers” category compared to the “decreasing activity, walkers”. SES was not associated with being in the “active, walker” group compared to the “decreasing, activity walker” group, indicating that the association between SES and physical activity multi-trajectory group is driven by the walking trajectory. While we did not see an association between birth order and individual trajectories, first-born females were less likely to be an “active, walker” than a “decreasing activity, walker.” This could be due to greater household responsibilities (perhaps having to help look after younger siblings) and therefore less time for play and sports in first-born female children.

Sedentary behavior has been viewed as displacing physical activity and as an independent health risk behavior [61]. Results from studies linking sedentary behavior and physical activity levels have been mixed [62]. We found that females who reported initially lower sedentary behavior reported more walking to and from school. The longer time spent walking may mean less time for sedentary activities once at home. The lack of relation between sedentary behavior and informal physical activity and organized sports in both males and females suggests that sedentary time was not displacing these activities.

Sleep and physical activity are thought to reciprocally influence each other through multiple physiological and psychosocial factors [63]. Regular MVPA has been associated with increased total sleep time and sleep quality in adolescents [64]. Daytime sleepiness resulting from insufficient sleep may lead to physical inactivity. We found that school-night sleep trajectory membership differed across walking to and from school trajectories in both males and females but did not differ across informal physical activity or organized sport trajectories. These findings suggest a possible time displacement of sleep for walking (such as having to get up early to walk to school). We found that school-night sleep trajectory membership also differed across sedentary behavior trajectory in both males and females, with those reporting more sedentary behavior reporting less sleep. This could be due to higher use of screen-based devices at night delaying bedtimes.

While multi-trajectory modeling was useful for summarizing physical activity behaviors in this population, we had limited success modeling physical activity together with sedentary behavior and sleep. We were able to create distinct multi-trajectory groups in males, but the primary distinctions between groups were in the physical activity domains. In females, the only distinction between groups was the walking to and from school trajectory. The goal of multi-trajectory modeling is to highlight heterogeneity in the linkage between trajectories of distinct behaviors that are thought to have common underlying etiological processes [45]. Our results indicate that physical activity, sedentary behavior and sleep may have different etiological processes in this urban South African population and should be looked at as independent health risk behaviors.

### Strengths and limitations

A major strength of our study was the use of longitudinal data throughout adolescence. Another strength of our study was the use of LCGA to group participants into common longitudinal trajectories of physical activity domains, sedentary behavior and sleep. The goal of LCGA is to model individual trajectories and is recommended when a single growth curve may not describe the variables of interest in the population adequately.

The use of LCGA comes with some limitations. In LCGA, class membership is inferred using a series of exploratory analyses to determine the best fitting model. The validity of LCGA may be questioned because model fit statistics may not adequately differentiate between a model with multiple latent classes and a single-class model with non-normal outcomes [65]. However, using multiple indicators of model fit strengthens the validity of our findings [66]. Both LCGA and multi-trajectory modeling assign class/group membership based on estimated posterior probabilities from a maximum likelihood analysis. Not all participants follow a trajectory/group perfectly and misclassification can occur [43, 45]. Our attempt to generate multi-trajectory groups that included physical activity, sedentary behavior and sleep had limited success, as there was not enough heterogeneity in the linkages between these behaviors in this population to identify clusters of individuals in terms of these outcomes together. The questionnaires changed slightly over time. All the measures we analyzed were self-reported, and we do not have data on intensity of informal physical activity or walking or on quality of sleep.

## Conclusion

The majority of this population of South Africa adolescents did not meet WHO recommendations for physical activity and demonstrated a decline over the adolescent period. In this population, trajectories of sedentary behavior and sleep were independent of physical activity.

## Additional files

Additional file 1: Questionnaire. (PDF 403 kb)

Additional file 2: Supplemental Figures S1-S3 and Supplemental Tables S1-S8 (DOCX 323 kb)

## Data Availability

The datasets used and/or analyzed during the current study are available from Dr. Shane Norris (shane.norris@wits.ac.za) on reasonable request.

## Abbreviations

BIC: Bayesian Information Criterion
BLRT: Bootstrap Likelihood Ratio Test
LGCA: Latent Growth Class Analysis
LMIC: Low- or Middle-Income Country
LMR-LRT: Lo-Medell-Rubin-Likelihood Ratio Test
MET: Metabolic equivalent
MVPA: Moderate or Vigorous Physical Activity
RRR: Relative Risk Ratio
SES: Socioeconomic status
WHO: World Health Organization
