# Supplementary material for: Longitudinal patterns of physical activity, sedentary behavior and sleep in urban South African adolescents, Birth-To-Twenty Plus cohort
Source: BMC Pediatr. 2019 Jul 18;19:241. doi: 10.1186/s12887-019-1619-z (PMC6639930; doi:10.1186/s12887-019-1619-z)
Supplement: Supplementary file 1 — Questionnaire. (PDF 403 kb) [file 12887_2019_1619_MOESM1_ESM.pdf]

University of the Witwatersrand
Department of Paediatrics and Child Health

BIRTH TO TWENTY BARA SITE: 13TH YEAR ADOLESCENT QUESTIONNAIRE

DATE : Day ☐☐ **Month** ☐☐ **Year** ☐☐☐☐

BTT ID NUMBER : ☐☐☐☐☐☐☐

BONE STUDY ID NUMBER : ☐☐☐

CONNECTEDNESS TO COMMUNITY, SCHOOL, PARENTS/CAREGIVERS

How much do you agree with the following statements?

	YES!	Yes	No	NO!
I feel safe in my neighbourhood				
There is crime in my neighbourhood				
If I had to move I would miss the neighbourhood I now live in				
There is drug selling in my neighbourhood				
There are fights in my neighbourhood				
There are opportunities in my school to talk to teachers one-to-one				
There are lots of opportunities in my school to get involved with sport, clubs and other activities during break and after school				
I feel safe at my school				
My teachers praise me when I work hard at school				
My school lets my parents know when I have done something well				
I feel very close to my mother / female caregiver				
I share my thoughts & feelings with my mother / caregiver				
I enjoy spending time with my mother				
I would want to be a person like my mother when I am her age				
I feel close to my father / another male caregiver				
I share my thoughts & feelings with my father / another male caregiver				
I enjoy spending time with my father / another male caregiver				
I would want to be a person like my father / another male caregiver when I am his age				
I can talk over important decisions with my mother or father or other caregiver				
If I had a personal problem, I could ask my mother or father or caregiver for help				
My parents / caregivers encourage me to do my best				
My parents / caregivers are proud of me when I do something well				
People in my family often insult and/or shout at each other				

PHYSICAL ACTIVITY

Activities at school

1. Do you attend physical education/games lessons at school?
   (Exercise classes supervised by a teacher during school time)

Yes=1	No=0	

2. How often classes are held & how long are classes?

Times / week	Hours / time

What are the three most frequent activities that you do during these classes?

Activities

3. Do your school teachers encourage you to participate in **physical activity**?

4. Do your parents encourage you to participate in **physical activity**?

**Y or N**	
**Y or N**	

5. Who (parent/caregiver or other) encourages you the most to participate in **physical activity?**

Informal activities

Do you engage in any physical activity during **school breaks** or **outside school**, for example riding a bike, playing in the street or yard? **NOT** activity as part of a sports team or club. Tick the three most frequent activities that you do, and time spent on each activity.

Activity	Mon	Tue	Wed	Thu	Fri	Sat	Sun
Riding a bike							
Playing with a ball							
Skipping							
Hop scotch							
Dibeke (tin game)							
Bhati (tennis ball game)							
Mgusha (panty hose game)							
Skateboarding/ roller-skating							
Other (list)							

Sedentary activities

Do you engage in any of the following activities before or after school, and if so, for how many hours?

Activity	Mon-Thur (hrs)	Fri-Sat (hrs)	Sun (hrs)
Watching TV & videos & movies			
Reading, drawing, homework			
Playing a musical instrument			
Playing video/ TV/ computer games			
Internet surfing			

What time do you go to bed on a school night?

Before 8pm	1	8 – 9:30pm	2	9:30 – 11pm	3	11-12:30pm	4	After 12:30pm	5	

What time do you go to bed on a non-school night (on a weekend or on holiday)?

Before 8pm	1	8 – 9:30pm	2	9:30 – 11pm	3	11-12:30pm	4	After 12:30pm	5	

What time do you wake up on a school morning?

Before 8am	1	8 – 9:30am	2	9:30 – 11am	3	11-12:30pm	4	After 12:30pm	5	

What time do you wake up on a non-school morning (on a weekend or on holiday)?

Before 8am	1	8 – 9:30am	2	9:30 – 11am	3	11-12:30pm	4	After 12:30pm	5	

Transport

How do you get to school and how long does it take to get there and back?

1. By car, bus, taxi, train etc.

Yes=1	No=2			
There:____________minutes				
Back:____________minutes				

2. Walking

Yes=1	No=2			
There:____________minutes				
Back:____________minutes				

When you walk, at what pace (how fast) do you usually walk?

At a pace, that makes me breathe much harder than normal	1	
At a pace that makes me breathe somewhat harder than normal	2	
At a pace where there is no change in my breathing	3	

3. Bicycle

Yes=1	No=2			
There:____________minutes				
Back:____________minutes				

When you cycle, at what pace (how fast) do you usually cycle?

At a pace, that makes me breathe much harder than normal	1	
At a pace that makes me breathe somewhat harder than normal	2	
At a pace where there is no change in my breathing	3	

NOTES:

Casual work

1. Not counting jobs around the house, do you sometimes work for **pay** (money) such as deliveries, gardening, cleaning, and looking after children?

**Y or N**	

2. If YES, what do you do? What are your duties?

____

____

____

3. When you do these kinds of jobs, how often do you usually work?
    a. About once a month
    b. A few times a month
    c. About once a week
    d. A few times a week
    e. 4 or more times a week
    f. Everyday

4. When you do these kinds of jobs, how many **hours** would you say you usually work per **week**?

EXTRA MURAL ACTIVITIES AT SCHOOL (LAST 12 MONTHS)

	How many months?	Prac/Wk	Hrs/Prac	Comp/Wk
Athletics (running)				
Athletics (other)				
Cricket				
Swimming				
Tennis				
Hockey				
Netball				
Rugby				
Soccer				
Badminton				
Basketball				
Ballet				
Cycling				
Dancing				
Gymnastics				
Judo / karate				
Squash				
Volleyball				
**Other**				

PRIVATE EXTRA MURAL ACTIVITIES (LAST 12 MONTHS)

	How many months?	Prac/Wk	Hrs/Prac	Comp/Wk
Athletics (running)				
Athletics (other)				
Cricket				
Swimming				
Tennis				
Hockey				
Netball				
Rugby				
Soccer				
Badminton				
Basketball				
Ballet				
Cycling				
Dancing				
Gymnastics				
Judo / karate				
Squash				
Volleyball				
**Other**				

SCHOOL REPORT

**Collected:** YES or NO	**School type:** PRIMARY or HIGH

Name of school:	
School address (NB - Suburb)	
Present Grade:	
Year of the report:	

LIFE EVENTS
Please tick all appropriate events that have happened in the **past 6 months**

Family moved to a new house	
Birth of a brother or sister	
Death of a parent	
Serious illness requiring hospitalization of brother or sister	
Marriage of parent to stepparent	
Divorce of a parent	
Changed schools	
Serious illness or accident requiring hospitalization for you	
Marital separation of parents	
Increase in arguments between parents	
Serious illness or accident requiring hospitalization of parent	
Death of a close friend	
Separation from close family for 2 weeks or more	
Death of a brother or sister	
Death of a grandparent	
Brother or sister leaving home	
Loss of job by parent	

RELIGION
1. How important is religion in your life?

Not at all	Somewhat	Very important	
0	1	2	☐

2. How often do you attend religious/spiritual services?

Less than once a year	0	
Once or twice a year	1	
Not every month, but at least every 2-3 months	2	
Not every week, but at least once a month	3	☐
At least once a week	4	

3. How often does your family pray together?

Never	0	
Seldom, can remember once or twice that we did	1	
Sometimes, at least once a month	2	
Often at least once a week	3	☐

TOBACCO, ALCOHOL, OTHER DRUGS

1. Have you ever tried or experimented with cigarette smoking even 1 or 2 puffs?

No 0 Yes 1

If YES,

How old were you when you first tried a cigarette?

2. During the past 30 days did you smoke cigarettes? No 0 Yes 1

If YES:

How often do you smoke?

1. Every day: How many cigarettes a day
2. A few times a week: How many cigarettes in a week
3. A few times a month: About how many cigarettes / month

IF YES: What BRAND of cigarettes do you smoke?

IF YES: Where do you usually smoke? (TICK ALL THAT APPLY)

At home	
At school	
At work	
At friends' houses	
At social events (parties)	
In public spaces (eg parks, outside shopping centres)	
Other	

IF YES: Where do you get the money to buy cigarettes? (TICK ALL THAT APPLY)

Use pocket money	
Receive payments for work	
Lift/steal money from people in the house	
Lift/steal cigarettes from people in the house	
Bum cigarettes off friends	
I buy loose cigarettes one at a time	
Remix stompies	
Other	

IF YES: have you ever tried to quit/stop smoking? No Yes

2. Do your parents/caregivers smoke?

None | Both Father/Male Caregiver only | Mother/Female Caregiver only | Don't know

4. Do you think you will smoke cigarettes when you are grown up?

No 0 Yes 1 Not Sure 2 ☐

5. If one of your best friends offered you a cigarette, would you smoke it?

Definitely Not | Probably Not | Probably Yes | Definitely Yes

6. When you see a man smoking what do you think of him?

7. When you see a woman smoking what do you think of her?

8. Do any of your closest friends smoke cigarettes?

None of them | Some of them | Most of them | All of them

9 (a). Has anyone in your family discussed the harmful effects of smoking with you?

No Yes

9 (b). During the past 6 months at school were you taught in any of your classes about the risks of cigarette smoking?

No Yes

10. When you watch TV, videos, or movies, how often do you see actors smoking?

I never watch TV, videos, or movies ☐

A lot ☐

Sometimes ☐

Never ☐

11. Have you ever tasted alcohol? (for other than religious purposes) No 0 Yes 1

If YES,
How old, in years, were you the first time you tasted alcohol?

12. Have you ever had an alcoholic drink? {A drink is defined as one can/bottle of beer one glass of wine, one tot of liquor, or one mixed drink}

No 0 Yes 1

If YES,
How old, in years, were you the first time you drank alcohol?

With whom have you drunk alcohol?

parents/guardians	No 0	Yes 1
brothers or sisters	No 0	Yes 1
friends	No 0	Yes 1
neighbours	No 0	Yes 1
alone	No 0	Yes 1

13. Do you drink alcohol now?

No 0 Yes 1 Sometimes 2

14. Do you **know** what the following drugs are? ---- Have you ever **used** the drug?

	Know		Used		Know	Used
Marijuana (weed, dagga, grass)	No 0	Yes 1	No 0	Yes 1		
Cocaine (coke/crack/rocks)	No 0	Yes 1	No 0	Yes 1		
LSD, Mushrooms, Acid	No 0	Yes 1	No 0	Yes 1		
Steroids	No 0	Yes 1	No 0	Yes 1		
Glue	No 0	Yes 1	No 0	Yes 1		
Ecstasy	No 0	Yes 1	No 0	Yes 1		
Mandrax (pinks)	No 0	Yes 1	No 0	Yes 1		

VIOLENCE

1. Have you ever carried a weapon for protection or for any other reason? No 0 Yes 1

If YES, what type?

- Gun 1
- Knife / blade 2
- Stick / knobkerrie 3
- Other 4

If Other, please describe

2. Do you know of a friend who has carried a weapon? No 0 Yes 1

If YES, what type?

- Gun 1
- Knife / blade 2
- Stick / knobkerrie 3
- Other 4

For what reason did they carry the weapon?

If Other, please describe

3.Have you ever been physically hurt by:

boyfriend / girlfriend	No 0	Yes 1
peers at school	No 0	Yes 1
family	No 0	Yes 1
strangers	No 0	Yes 1
others (specify)	No 0	Yes 1

4. Have you ever been in trouble with the law? No 0 Yes 1

If YES, please explain

SEXUAL KNOWLWEDGE AND EXPERIENCE

1. Have you ever had a health education class at **school** that included sex education? No 0 Yes 1

If YES,

In what grade [ ]

Was this useful to you? No 0 Yes 1 Somewhat 2

2. Have you ever talked about AIDS/HIV infection with your parents or other adults in your family No 0 Yes 1 Not Sure 2

3. Have you ever discussed birth control/family planning/contraceptive methods with: (Please answer EACH item.)

a. Your parents / guardians No 0 Yes 1

b. Your friends No 0 Yes 1

c. Your teacher, counsellor or coach No 0 Yes 1

d. Your doctor or clinic nurse No 0 Yes 1

e. Others No 0 Yes 1

If YES, please describe

[ ]

4. Do you know what it means to "have sex" with someone? No 0 Yes 1

If YES, continue

If NO SKIP section

5. Have you ever engaged in foreplay or heavy petting (i.e. not going "all the way")? No 0 Yes 1 Not Sure 2

If YES,

How old were you in years when this first happened?

IF NO SKIP section

[ ]

How old was your partner or if you have done it more than once,

How old was your first partner? [ ]

How old was your most recent partner? [ ]

Was this something you wanted to participate in? No 0 Yes 1 Not Sure 2

6. Have you ever had sex (made love, gone all the way, penis inserted in vagina or anus)? No 0 Yes 1

IF YES, continue

How old were you in years when you had sex the first time? [ ]

Was this something you wanted to participate in? No 0 Yes 1

What sex/gender was the person you had sex with Male 1 Female 2

How old was your partner or if you have done it more than once,

How old was your first partner? [ ]

How old was your most recent partner? [ ]

7. Have you ever had sex or been touched in private areas against your will? No 0 Yes 1 Don't Know 2

If YES: How old was the person who did this to you [ ]

What sex/gender was the person? Male 1 Female 2

Have you ever had sex or touched another person in private areas against his/her will? No 0 Yes 1 Don't Know 2

If YES: How old was the person you did it to? [ ]

FRIENDS

1. How many close friends do you have who are boys? [ ]
2. How many close friends do you have who are girls? [ ]
3. Are **most** of these close friends **(Select one only)**
    a. In your grade
    b. In a higher grade
    c. In a lower grade
    d. Not in school
    e. Don't have any close friends [ ]
4. How often do you feel lonely and wish you had more friends? (**Select one only**)
    a. Often
    b. Sometimes
    c. Hardly ever [ ]

ROSENBERG SELF-ESTEEM SCALE

	A lot like me	A bit like me	Not very like me	Not at all like me
1. On the whole, I am satisfied with myself	1	2	3	4
2. At times I think I am no good at all	1	2	3	4
3. I feel that I have a number of good qualities	1	2	3	4
4. I am able to do things as well as most other people	1	2	3	4
5. I feel I do not have much to be proud of	1	2	3	4
6. I certainly feel useless at times	1	2	3	4
7. I feel that I am a person of worth, at least on an equal plane with others	1	2	3	4
8. I wish I could have more respect for myself	1	2	3	4
9. All in all, I am inclined to feel that I am a failure	1	2	3	4
10. I take a positive attitude towards myself	1	2	3	4

SCHOOL RATING

1. How would you rate your school?

   Excellent | Good | Ok | Not too good | Poor

2. How would you rate your own performance at school?

   Excellent | Good | Ok | Not too good | Poor

3. Are you bullied by other children at school?

   Regularly | Sometimes | Never

4. Have you ever been part of a group which bullies other children?

   Regularly | Sometimes | Never

BODY ESTEEM SCALE FOR ADOLESCENTS

	Never	Seldom	Sometimes	Often	Always
1. I like what I look like in pictures	0	1	2	3	4
2. Other people consider me good looking	0	1	2	3	4
3. I'm proud of my body	0	1	2	3	4
4. I'm preoccupied with trying to change my body weight	0	1	2	3	4
5. I like what I see when I look in the mirror	0	1	2	3	4
6. There are lots of things I'd like to change about my looks if I could	0	1	2	3	4
7. I am satisfied with my weight	0	1	2	3	4
8. I wish I looked better	0	1	2	3	4
9. I really like what I weigh	0	1	2	3	4
10. I wish I looked like someone else	0	1	2	3	4
11. People my own age like my looks	0	1	2	3	4
12. My looks upset me	0	1	2	3	4
13. I'm as nice looking as most people	0	1	2	3	4
14. I'm pretty happy about the way I look	0	1	2	3	4
15. I feel I weigh the right amount for my height	0	1	2	3	4
16. I feel ashamed of how I look	0	1	2	3	4
17. Weighing myself depresses me	0	1	2	3	4
18. My weight makes me unhappy	0	1	2	3	4
19. I worry about the way I look	0	1	2	3	4
20. I think I have a good body	0	1	2	3	4
21. I'm looking as nice as I'd like to	0	1	2	3	4

HIV / AIDS

1. Do you know anyone who is suffering from HIV/AIDS? No 0

Yes 1

If YES, is that person

A family member 1

A friend 2

Someone in the neighbourhood 3

Someone you have heard about / elsewhere 4

2. Do you know anyone who has died of HIV/AIDS? No 0

Yes 1

If YES, is that person

A family member 1

A friend 2

Someone in the neighbourhood 3

Someone you have heard about / elsewhere 4

3. Are you having to take care of or financially support anyone who now has HIV/AIDS or because someone else has AIDS or died of AIDS?

No Yes

PARENT WHEREABOUTS

1. Are you living with both your parents? No 0 Yes 1

If No,

Do you live with your mother No 0 Yes 1

If not living with mother,

Since what age in years have you not lived with your mother?

Do you see your mother? No 0 Yes 1

If Yes, how often

Never 0	See her very seldom 1	More than once a year 2	More than once a month 3	More than once a week 4

☐

Do you live with your father No 0 Yes 1

If not living with father,

Since what age in years have you not lived with your father?

☐☐

Do you see your father? No 0 Yes 1

If Yes, how often

Never 0	See him very seldom 1	More than once a year 2	More than once a month 3	More than once a week 4

☐

BELIEFS ABOUT OBESE PERSONS SCALE (BAOP)

Please mark each statement below according to how much you agree or disagree with it.

No	Question	**YES!**	**Yes**	**No**	**NO!**
1	Overweight teenagers eat because they don't get love or attention				
2	In many cases overweight teenagers are fat because of a biological or health problem.				
3	Over-weight teenagers get fat because of overeating.				
4	Most overweight teenagers get fat because they don't get enough exercise.				
5	Most overweight teenagers eat more than non-overweight teenagers.				
6	The majority of overweight teenagers have bad eating habits that cause them to be fat.				
7	Being fat is not caused by a lack of will power.				
8	Teenagers can be addicted to food as others are addicted to drugs and alcohol and teenagers that are addicted to food usually become overweight/fat.				

DISABILITY RATING TASK
Place the cards in order from the child you are most likely to be friends with to the one you least likely to be friends with.

Teenager who is blind	
Teenager with crutches and leg brace	
Teenager sitting in a wheelchair	
Teenager with no hand	
Child with facial disfigurement (hair lip)	
A teenager who is an Albino	
A fat teenager	
A teenager who comes from a poor family	

WEIGHT CHANGE ATTEMPTS

1. Have you tried to **lose weight** during the past year?

Yes	No

2. If yes, what was the **most important** reason (**mark only one**)?

It is healthy	
I want to look better	
My clothes were too tight	
I am too fat compared to my friends	
I am unhappy with myself	
I have a dreams of being a model or movie/TV star	
Any other reason, specify	

3. If you did try to **lose weight**, describe all the methods you have tried. Include any information on diet, exercise, pills or anything else that you have tried.

1.<br>2.<br>3.<br>4.<br>5.

4. Did you try to **build more muscles** or grow bigger during the past year?

Yes	No

5. If yes, what was the most important reason (mark only one)?

It is healthy	
I want to look better	
I have too little muscles compared to my friends	
I am unhappy with myself	
I have a dreams of being a model or movie/TV star	
Any other reason, specify	

6. If you did **try to build more muscles**, describe all the methods you have tried. Include any information on diet, exercise, pills or anything else that you have tried.

1.<br>2.<br>3.<br>4.<br>5.

ACCULTURATION

	1st	2nd	3rd
My 3 favourite musicians are?			
My 3 favourite foods are?			
The 3 people that are my role models are?			
The 3 people who have a body that I would like to have are?			
My 3 favourite sports stars are?			
My 3 favourite celebrities are?			

EATING ATTITUDES TEST

Please make a cross under the column which applies best to the way you feel next to each statement.

	Always	Very often	Often	Sometimes	Seldom	Never
1. I am terrified *(very scared)* about being overweight						
2. I avoid eating *(try not to eat)* when I am hungry						
3. I find myself preoccupied with food *(think about food a lot)*						
4. I have gone on eating binges *(a lot of food in a short time)* where I feel that I may not be able to stop						
5. I cut my food into small pieces						
6. I am aware of the calorie/ *kilojoule (energy)* content of foods that I eat						
7. I particularly avoid foods with a high carbohydrate *(starch)* content *such* as bread, potatoes, rice *and pap*						
8. I feel that others would prefer *(like it)* if I ate more						
9. I vomit *(bring up food / throw up)* after I have eaten						
10. I feel extremely guilty (*I've done wrong)* after eating						
11. I am preoccupied with a desire to be thinner *(think about being thinner a lot)*						
12. I think about burning up calories/ kilojoules *(energy)* when I exercise						
13. Other people think I am too thin						
14. I am preoccupied with the thought of having fat on my body *(think about having fat on my body a lot)*						
15. I take longer than other people to eat my meals *(food)*						
16. I avoid *(try not to eat)* foods with sugar in them						
17. I eat "diet" foods *(special foods to lose weight)*						
18. I feel that food controls my life						
19. I display self control around food (*I can control my eating if there is a lot of food available)*						
20. I feel that others put pressure on me to eat						
21. I give too much time and thought to food						
22. I feel uncomfortable *(not good)* after eating sweets						
23. I engage in dieting behaviour *(try to lose weight)*						
24. I like my stomach to be empty *(I like the feeling)*						
25. I enjoy trying new rich (*creamy/ fatty*) foods						
26. I have the impulse *(need)* to vomit after meals						

ADOLESCENT MEASUREMENTS

SECTION A:

- STANDING HEIGHT: (mm)
- SITTING HEIGHT: (mm)
- WEIGHT: (kg)
- WAIST CIRCUMFERENCE: (mm)
- HIP CIRCUMFERENCE: (mm)

SECTION B: BLOOD PRESSURE

- SYSTOLIC BP
- DIASTOLIC BP
- PULSE
- TIME OF BP   h

SECTION C: **DXA SCANS COMPLETED**

(Whole body, Hip, Spine)

SECTION D: COLLECTION OF FASTING SPECIMENS

HAVE YOU HAD ANYTHING TO EAT SINCE LAST NIGHT?	*Y*	*N*
1ST URINE SAMPLE.	*Y*	*N*
ULE URINE TEST	*Y*	*N*
ROUTINE BLOOD	*Y*	*N*
OGTT	*Y*	*N*
DNA (If applicable)	*Y*	*N*

SECTION I: DIETARY INTAKE

24 Hour Dieatry Recall Questionnaire	*Y*	*N*

SECTION J: PUBERTAL ASSESSMENT

Pubertal assessment Questionnaire	*Y*	*N*
