# Supplementary material for: Longitudinal patterns of physical activity, sedentary behavior and sleep in urban South African adolescents, Birth-To-Twenty Plus cohort
Source: BMC Pediatr. 2019 Jul 18;19:241. doi: 10.1186/s12887-019-1619-z (PMC6639930; doi:10.1186/s12887-019-1619-z)
Supplement: Supplementary file 2 — Supplemental Figures S1-S3 and Supplemental Tables S1-S8 (DOCX 323 kb) [file 12887_2019_1619_MOESM2_ESM.docx]

Supplemental Table 1. Latent Class Growth Analysis model fit for physical activity by domain and sex, Birth-to-Twenty+^1^

|  | **Informal Activity** | | | | | | | |  |  |  |  |
| --- | --- | --- | --- | --- | --- | --- | --- | --- | --- | --- | --- | --- |
| Sex | Latent classes | BIC | LMR-LRT  (p-value) | BLRT  (p-value) | Convergence | Entropy value | Log likelihood | Number of parameters | class 1 | class 2 | class 3 | class 4 |
| Males | 1 | 48344 | N/A | N/A | Yes | N/A | -24149 | 7 | 100% |  |  |  |
|  | **2** | **48180** | **0.11** | **0.00** | **Yes** | **0.92** | **-24058** | **10** | **7%** | **93%** |  |  |
|  | 3 | 48120 | 0.02 | 0.00 | Yes | 0.88 | -24018 | 13 | 7% | 87% | 7% |  |
| Females | 1 | 51706 | N/A | N/A | N/A | Yes | -25830 | 7 | 100% |  |  |  |
|  | **2** | **51285** | **0.23** | **0.24** | **Yes** | **0.99** | **-25610** | **10** | **5%** | **95%** |  |  |
|  | 3 | 51035 | 0.49 | 0.00 | Yes | 0.99 | -25474 | 13 | 92% | 2% | 6% |  |
|  | **Physical Education** | | | | | | | |  |  |  |  |
| Sex | Latent classes | BIC | LMR-LRT  (p-value) | BLRT  (p-value) | Convergence | Entropy value | Log likelihood | Number of parameters | class 1 | class 2 | class 3 | class 4 |
| Males | **1** | **38424** | **N/A** | **N/A** | **Yes** | **N/A** | **-19186** | **8** | **100%** |  |  |  |
|  | 2 | 38167 | 0.40 | 0.00 | Yes | 0.99 | -19048 | 11 | 97% | 3% |  |  |
|  | 3 | 37992 | 0.24 | 0.00 | Yes | 0.99 | -18950 | 14 | 3% | 4% | 93% |  |
| Females | **1** | **41917** | **N/A** | **N/A** | **Yes** | **N/A** | **-20932** | **8** | **100%** |  |  |  |
|  | 2 | 41646 | 0.00 | 0.00 | Yes | 0.99 | -20787 | 11 | 3% | 97% |  |  |
|  | 3 | 41487 | 0.42 | 0.00 | Yes | 0.97 | -20697 | 14 | 2% | 91% | 7% |  |
|  | **Organized Sports** | | | | | | | |  |  |  |  |
| Sex | Latent classes | BIC | LMR-LRT  (p-value) | BLRT  (p-value) | Convergence | Entropy value | Log likelihood | Number of parameters | class 1 | class 2 | class 3 | class 4 |
| Males | 1 | 55381 | N/A | N/A | Yes | N/A | -27664 | 8 | 100% |  |  |  |
|  | 2 | 55063 | 0.03 | 0.00 | Yes | 0.88 | -27496 | 11 | 15% | 85% |  |  |
|  | **3** | **55015** | **0.18** | **0.00** | **Yes** | **0.85** | **-27462** | **14** | **81%** | **11%** | **8%** |  |
|  | 4 | 54991 | 0.09 | 0.00 | Yes | 0.90 | -27440 | 17 | 7% | 76% | 3% | 14% |
| Females | 1 | 99432 | N/A | N/A | Yes | N/A | -49687 | 8 | 100% |  |  |  |
|  | **2** | **98261** | **0.06** | **0.00** | **Yes** | **0.97** | **-49091** | **11** | **93%** | **7%** |  |  |
|  | 3 | 97619 | 0.31 | 0.00 | Yes | 0.97 | -48759 | 14 | 91% | 7% | 2% |  |
|  | **Walking to and from School** | | | | | | | |  |  |  |  |
| Sex | Latent classes | BIC | LMR-LRT  (p-value) | BLRT  (p-value) | Convergence | Entropy value | Log likelihood | Number of parameters | class 1 | class 2 | class 3 | class 4 |
| Males | 1 | 49286 | N/A | N/A | Yes | N/A | -24617 | 8 | 100% |  |  |  |
|  | **2** | **48792** | **0.03** | **0.00** | **Yes** | **0.82** | **-24360** | **11** | **81%** | **19%** |  |  |
|  | 3 | 48650 | 0.20 | 0.00 | Yes | 0.78 | -24279 | 14 | 3% | 31% | 66% |  |
|  | 4 | 48592 | 0.19 | 0.00 | Yes | 0.70 | -24241 | 17 | 5% | 44% | 10% | 41% |
| Females | 1 | 55986 | N/A | N/A | Yes | N/A | -27967 | 8 | 100% |  |  |  |
|  | **2** | **55406** | **0.36** | **0.00** | **Yes** | **0.79** | **-27667** | **11** | **75%** | **25%** |  |  |
|  | 3 | 55270 | 0.03 | 0.00 | Yes | 0.85 | -27589 | 14 | 1% | 72% | 27% |  |

^1^Bold text indicates selected model

Abbreviations: BIC: Bayesian Information Criterion; LMR-LRT: Lo-Medell-Rubin-Likelihood Ratio Test; BLRT: Bootstrap Likelihood Ratio Test

Supplemental Table 2. Latent Class Growth Analysis model fit for sedentary behavior and sleep by sex, Birth-to-Twenty+^1^

|  | **Sedentary Behavior** | | | | | | | |  |  |  |  |  |
| --- | --- | --- | --- | --- | --- | --- | --- | --- | --- | --- | --- | --- | --- |
| Sex | Latent classes | BIC | LMR-LRT  (p-value) | BLRT  (p-value) | Convergence | Entropy value | Log likelihood | Number of parameters | class 1 | class 2 | class 3 | class 4 | class 5 |
| Males | 1 | 59096 | N/A | N/A | Yes | N/A | -29522 | 8 | 100% |  |  |  |  |
|  | 2 | 58892 | 0.00 | 0.00 | Yes | 0.70 | -29410 | 11 | 23% | 77% |  |  |  |
|  | **3** | **58815** | **0.00** | **0.00** | **Yes** | **0.78** | **-29362** | **14** | **13%** | **76%** | **11%** |  |  |
|  | 4 | 58818 | 0.56 | 0.05 | Yes | 0.73 | -29354 | 17 | 4% | 9% | 18% | 69% |  |
|  | 5 | 58820 | 0.40 | 0.13 | Yes | 0.73 | -29345 | 20 | 19% | 4% | 67% | 9% | 2% |
| Females | 1 | 65539 | N/A | N/A | Yes | N/A | -32743 | 8 |  |  |  |  |  |
|  | **2** | **65361** | **0.05** | **0.00** | **Yes** | **0.90** | **-32644** | **11** | **91%** | **9%** |  |  |  |
|  | 3 | 65242 | 0.00 | 0.00 | Yes | 0.66 | -32574 | 14 | 59% | 7% | 34% |  |  |
|  | 4 | 65233 | 0.74 | 0.00 | Yes | 0.68 | -32560 | 17 | 5% | 4% | 54% | 37% |  |
|  | 5 | 65223 | 0.01 | 0.01 | Yes | 0.75 | 32546 | 20 | 8% | 58% | 2% | 32% | 0% |
|  | **School-night Sleep** | | | | | | | |  |  |  |  |  |
| Sex | Latent classes | BIC | LMR-LRT  (p-value) | BLRT  (p-value) | Convergence | Entropy value | Log likelihood | Number of parameters | class 1 | class 2 | class 3 | class 4 | class 5 |
| Males | 1 | 26288 | N/A | N/A | Yes | N/A | -13121 | 7 | 100% |  |  |  |  |
|  | 2 | 26125 | 0.000 | 0.000 | Yes | 0.56 | -13030 | 10 | 60% | 40% |  |  |  |
|  | 3 | 26038 | 0.000 | 0.000 | Yes | 0.73 | -12976 | 13 | 10% | 25% | 65% |  |  |
|  | **4** | **26024** | **0.032** | **0.000** | **Yes** | **0.72** | **-12960** | **16** | **5%** | **9%** | **54%** | **31%** |  |
|  | 5 | 26032 | 0.035 | 0.000 | Yes | 0.76 | -12954 | 19 | 8% | 58% | 5% | 29% |  |
|  |  |  |  |  |  |  |  |  |  |  |  |  |  |
| Females | 1 | 28374 | N/A | N/A | Yes | N/A | -14164 | 7 | 100% |  |  |  |  |
|  | 2 | 28144 | 0.000 | 0.000 | Yes | 0.60 | -14039 | 10 | 58% | 42% |  |  |  |
|  | 3 | 28074 | 0.087 | 0.000 | Yes | 0.69 | -13994 | 13 | 8% | 38% | 54% |  |  |
|  | 4 | 28054 | 0.280 | 0.000 | Yes | 0.67 | -13974 | 16 | 21% | 28% | 3% | 49% |  |
|  | **Weekend Sleep** | | | | | | | |  |  |  |  |  |
| Sex | Latent classes | BIC | LMR-LRT  (p-value) | BLRT  (p-value) | Convergence | Entropy value | Log likelihood | Number of parameters | class 1 | class 2 | class 3 | class 4 | class 5 |
| Males | 1 | 29625 | N/A | N/A | Yes | N/A | -14790 | 7 | 100% |  |  |  |  |
|  | 2 | 29512 | 0.02 | 0.00 | Yes | 0.46 | -14723 | 10 | 47% | 53% |  |  |  |
|  | **3** | **29456** | **0.34** | **0.00** | **Yes** | **0.72** | **-14686** | **13** | **19%** | **73%** | **8%** |  |  |
|  | 4 | 29446 | 0.08 | 0.00 | Yes | 0.66 | -14671 | 16 | 2% | 10% | 32% | 56% |  |
| Females | 1 | 32787 | N/A | N/A | Yes | N/A | -16371 | 7 | 100% |  |  |  |  |
|  | 2 | 32652 | 0.00 | 0.00 | Yes | 0.48 | -16293 | 10 | 47% | 53% |  |  |  |
|  | 3 | 32622 | 0.03 | 0.00 | Yes | 0.63 | -16268 | 13 | 66% | 19% | 15% |  |  |
|  | **4** | **32627** | **0.40** | **0.00** | **Yes** | **0.62** | **-16261** | **16** | **37%** | **7%** | **47%** | **9%** |  |

^1^Bold text indicates selected model

Abbreviations: BIC: Bayesian Information Criterion; LMR-LRT: Lo-Medell-Rubin-Likelihood Ratio Test; BLRT: Bootstrap Likelihood Ratio Test

Supplemental Table 3. Sedentary behavior trajectory class membership across physical activity trajectories in males, Birth-to-Twenty+

|  |  | Sedentary behavior (%) | | | |
| --- | --- | --- | --- | --- | --- |
|  | Trajectory class | 1 | 2 | 3 | p-value |
| Informal activity (%) | 1 | 79 | 12 | 10 | 0.38 |
|  | 2 | 70 | 14 | 16 |  |
|  |  |  |  |  |  |
| Organized sports (%) | 1 | 78 | 12 | 10 | 0.28 |
|  | 2 | 82 | 5 | 12 |  |
|  | 3 | 75 | 18 | 7 |  |
|  |  |  |  |  |  |
| Walking to and from school (%) | 1 | 77 | 12 | 11 | 0.77 |
|  | 2 | 81 | 10 | 9 |  |

Supplemental Table 4. Sedentary behavior trajectory class membership across physical activity trajectories in females, Birth-to-Twenty+

|  | Trajectory Class | Sedentary behavior (%) | | |
| --- | --- | --- | --- | --- |
|  |  | 1 | 2 | p-value |
| Informal activity (%) | 1 | 92 | 8 | 0.97 |
|  | 2 | 92 | 8 |  |
|  |  |  |  |  |
| Organized sports (%) | 1 | 92 | 8 | 0.28 |
|  | 2 | 89 | 11 |  |
|  |  |  |  |  |
| Walking to and from school (%) | 1 | 91 | 9 | 0.04 |
|  | 2 | 96 | 4 |  |

Supplemental Table 5. Sleep trajectory class membership across physical activity trajectories in males, Birth-to-Twenty+

|  |  | School-night sleep (%) | | | | | Weekend sleep (%) | | |  |
| --- | --- | --- | --- | --- | --- | --- | --- | --- | --- | --- |
|  |  | 1 | 2 | 3 | 4 | p-value | 1 | 2 | 3 | p-value |
| Informal activity (%) | 1 | 56 | 31 | 8 | 4 | 0.8 | 78 | 16 | 6 | 0.66 |
|  | 2 | 52 | 34 | 7 | 7 |  | 77 | 14 | 9 |  |
|  |  |  |  |  |  |  |  |  |  |  |
| Organized sports (%) | 1 | 55 | 32 | 9 | 4 | 0.74 | 78 | 16 | 6 | 0.21 |
|  | 2 | 63 | 29 | 5 | 3 |  | 77 | 16 | 3 |  |
|  | 3 | 59 | 25 | 9 | 7 |  | 70 | 16 | 16 |  |
|  |  |  |  |  |  |  |  |  |  |  |
| Walking to and from school (%) | 1 | 55 | 33 | 7 | 5 | 0.05 | 81 | 14 | 5 | 0.001 |
|  | 2 | 58 | 25 | 14 | 3 |  | 64 | 25 | 10 |  |
|  |  |  |  |  |  |  |  |  |  |  |
| Sedentary behavior (%) | 1 | 60 | 26 | 10 | 3 | p<0.001 | 77 | 18 | 4 | p<0.001 |
|  | 2 | 43 | 49 | 0 | 8 |  | 79 | 4 | 17 |  |
|  | 3 | 38 | 51 | 3 | 9 |  | 78 | 13 | 9 |  |

Supplemental Table 6. Sleep trajectory class membership across physical activity trajectories in females, Birth-to-Twenty+

|  |  | School-night sleep (%) | | | | Weekend sleep (%) | | |  |  |
| --- | --- | --- | --- | --- | --- | --- | --- | --- | --- | --- |
|  | Trajectory Class | 1 | 2 | 3 | p-value | 1 | 2 | 3 | 4 | p-value |
| Informal activity (%) | 1 | 57 | 36 | 7 | 0.55 | 51 | 36 | 8 | 5 | 0.23 |
|  | 2 | 49 | 40 | 11 |  | 43 | 32 | 14 | 11 |  |
|  |  |  |  |  |  |  |  |  |  |  |
| Organized sports (%) | 1 | 57 | 35 | 7 | 0.3 | 51 | 36 | 8 | 6 | 0.87 |
|  | 2 | 49 | 44 | 7 |  | 54 | 35 | 7 | 4 |  |
|  |  |  |  |  |  |  |  |  |  |  |
| Walking to and from school (%) | 1 | 59 | 33 | 8 | 0.001 | 51 | 35 | 8 | 6 | 0.86 |
|  | 2 | 48 | 47 | 11 |  | 50 | 38 | 8 | 4 |  |
|  |  |  |  |  |  |  |  |  |  |  |
| Sedentary behavior (%) | 1 | 56 | 37 | 7 | 0.002 | 51 | 36 | 7 | 6 | 0.03 |
|  | 2 | 63 | 20 | 17 |  | 51 | 30 | 17 | 2 |  |

Supplemental Table 7. Multi-trajectory analysis model fit statistics for physical activity domains by sex, Birth-to-Twenty+^1^

| Sex | # of multi-trajectory groups | BIC | Group1 | Group 2 | Group 3 | Group 4 | Group 5 |
| --- | --- | --- | --- | --- | --- | --- | --- |
| Males | 1 | -56262.97 | 100% |  |  |  |  |
|  | 2 | -56038.56 | 28% | 72% |  |  |  |
|  | **3** | **-55898.43** | **23%** | **29%** | **49%** |  |  |
|  | 4 | variance matrix is nonsymmetric or highly singular | | | |  |  |
|  | 5 | variance matrix is nonsymmetric or highly singular | | | |  |  |
|  |  |  |  |  |  |  |  |
| Females | 1 | -50231.71 | 100% |  |  |  |  |
|  | 2 | -49912.22 | 37% | 63% |  |  |  |
|  | **3** | **-49783.08** | **31%** | **17%** | **51%** |  |  |
|  | 4 | variance matrix is nonsymmetric or highly singular | | | |  |  |
|  | 5 | variance matrix is nonsymmetric or highly singular | | | |  |  |

^1^Bold text indicates selected model

Abbreviations: BIC: Bayesian Information Criterion

Supplemental Table 8. Multi-trajectory analysis model fit statistics for physical activity domains, sedentary behavior and sleep by sex, Bt20+ Cohort^1^

| Sex | # of multi-trajectory groups | BIC | Group 1 | Group 2 | Group 3 | Group 4 |
| --- | --- | --- | --- | --- | --- | --- |
| Male | 1 | -90615.82 | 100% |  |  |  |
|  | 2 | -90214.94 | 67% | 33% |  |  |
|  | 3 | -90121.73 | 31% | 62% | 75% |  |
|  | **4** | **-90016.36** | **23%** | **42%** | **29%** | **7%** |
|  | 5 | variance matrix is nonsymmetric or highly singular | | | |  |
|  |  |  |  |  |  |  |
| Female | 1 | -88704.97 | 100% |  |  |  |
|  | **2** | **-88212.35** | **56%** | **44%** |  |  |
|  | 3 | variance matrix is nonsymmetric or highly singular | | | |  |
|  | 4 | variance matrix is nonsymmetric or highly singular | | | |  |
|  | 5 | variance matrix is nonsymmetric or highly singular | | | |  |

^1^Bold text indicates selected model

Abbreviations: BIC: Bayesian Information Criterion

Supplemental Figure 1. Flow chart depicting the final sample of eligible participants included in the analysis of physical activity patterns in adolescence, Birth to Twenty Plus Cohort, Johannesburg, South Africa

Supplemental Figure 2. Multi-trajectory groups of physical activity domains through adolescence, by sex, Birth to Twenty Plus Cohort, Johannesburg, South Africa


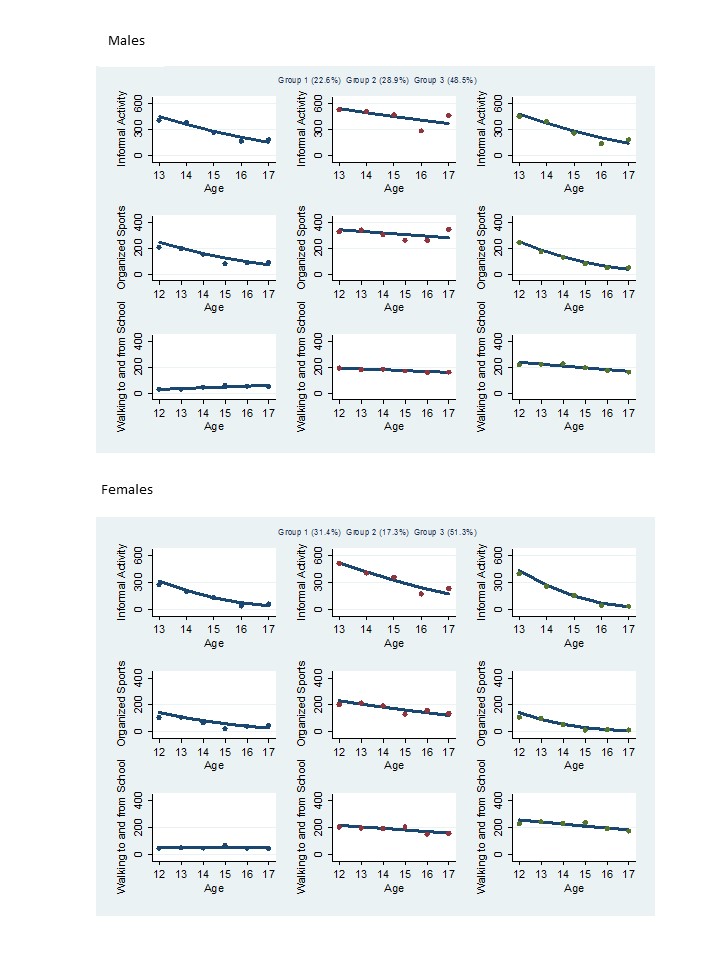


Supplemental Figure 3. Multi-trajectory groups of movement behavior domains through adolescence, by sex, Birth to Twenty Plus Cohort, Johannesburg, South Africa

Females

Males

Group 1 (56%) Group 2 (44%)

Group 1 (22%) Group 2 (42% Group 3 (29%) Group 4 (7%)
